# Acute Periflexural Purpura as a Revealing Sign of Parvovirus B19 Infection in a Child

**DOI:** 10.7759/cureus.107387

**Published:** 2026-04-20

**Authors:** Zineb Mernissi, Maryem Aboudourib, Layla Bendaoud, Ouafa Hocar, Said Amal

**Affiliations:** 1 Department of Dermatology, Mohammed VI University Hospital, Biosciences Laboratory, Faculty of Medicine and Pharmacy, Marrakech, MAR

**Keywords:** b19 parvovirus infection, b19 parvovirus serology, pediatric purpura, periflexural eruption, petechial rash

## Abstract

Purpura is a frequent dermatologic manifestation in children, arising from a broad spectrum of conditions ranging from benign viral infections to systemic disorders. Among viral causes, parvovirus B19 has been reported to occasionally induce purpuric eruptions in children, sometimes mimicking more severe hematologic or inflammatory disorders. Parvovirus B19 is classically associated with erythema infectiosum, but it can occasionally manifest with atypical cutaneous eruptions, including purpuric lesions in unusual distributions. We report the case of an 11-year-old child with no prior medical history who presented to the pediatric emergency department with a purpuric rash that had been evolving over a week and mild fever. Examination revealed purpuric petechial lesions, predominantly affecting the periflexural region, including the axillary and elbow folds, accompanied by a maculo-petechial enanthem. Laboratory studies showed leukopenia and neutropenia with mildly elevated C-reactive protein. Serology confirmed a primary parvovirus B19 infection based on positive IgM antibodies. The patient’s condition resolved spontaneously within one week with symptomatic treatment. This case highlights that parvovirus B19 infection may present as acute periflexural purpura, broadening the known clinical presentation of the infection. Awareness of these atypical presentations is essential to prevent unnecessary diagnostic procedures and inappropriate therapies. Serological testing or PCR may be crucial for diagnosis and guiding patient management, ensuring accurate assessment and appropriate symptomatic care.

## Introduction

Purpura is a common dermatologic manifestation in children and may arise from a wide spectrum of conditions ranging from benign causes to serious systemic diseases, including vasculitis, hematologic disorders, and infections. Clinically, purpura results from the extravasation of red blood cells into the skin and mucous membranes and may present as petechiae, ecchymoses, or palpable purpura. Due to the broad etiologic diagnosis and the potential severity of some underlying causes, accurate evaluation is essential in pediatric patients presenting with purpuric eruptions [1].

Among infectious causes, viral agents are well-recognized triggers of purpuric rashes in children. Several viruses, including parvovirus B19, enterovirus, Epstein-Barr virus, and adenovirus, have been associated with purpuric eruptions through mechanisms such as immune-mediated vascular injury, transient bone marrow suppression, or platelet dysfunction [2]. These eruptions may clinically resemble primary vasculitic or hematologic disorders, sometimes leading to diagnostic uncertainty and unnecessary investigations.

Parvovirus B19, a virus of the *Parvoviridae* family, is best known as the causative agent of erythema infectiosum (fifth disease), a common childhood exanthem characterized by the classic “slapped-cheek” facial erythema followed by a reticular rash on the trunk and extremities [3]. In addition to the classic exanthem, several atypical cutaneous manifestations have been described, including papular-purpuric “gloves and socks” syndrome, petechial or purpuric eruptions, and periflexural rashes that may mimic inflammatory or vasculitic dermatoses [4,5]. Recognition of these atypical presentations is important in clinical practice, particularly in pediatric emergency settings, where purpuric eruptions may raise concern for severe conditions such as IgA vasculitis or meningococcal infection. Awareness of viral etiologies, including parvovirus B19, can facilitate appropriate diagnostic evaluation and avoid unnecessary invasive procedures.

We report the case of an 11-year-old child presenting with acute periflexural purpura as the initial manifestation of parvovirus B19 infection, highlighting the importance of considering viral infections in the differential diagnosis of purpuric eruptions in children.

## Case presentation

**Figure 1:**
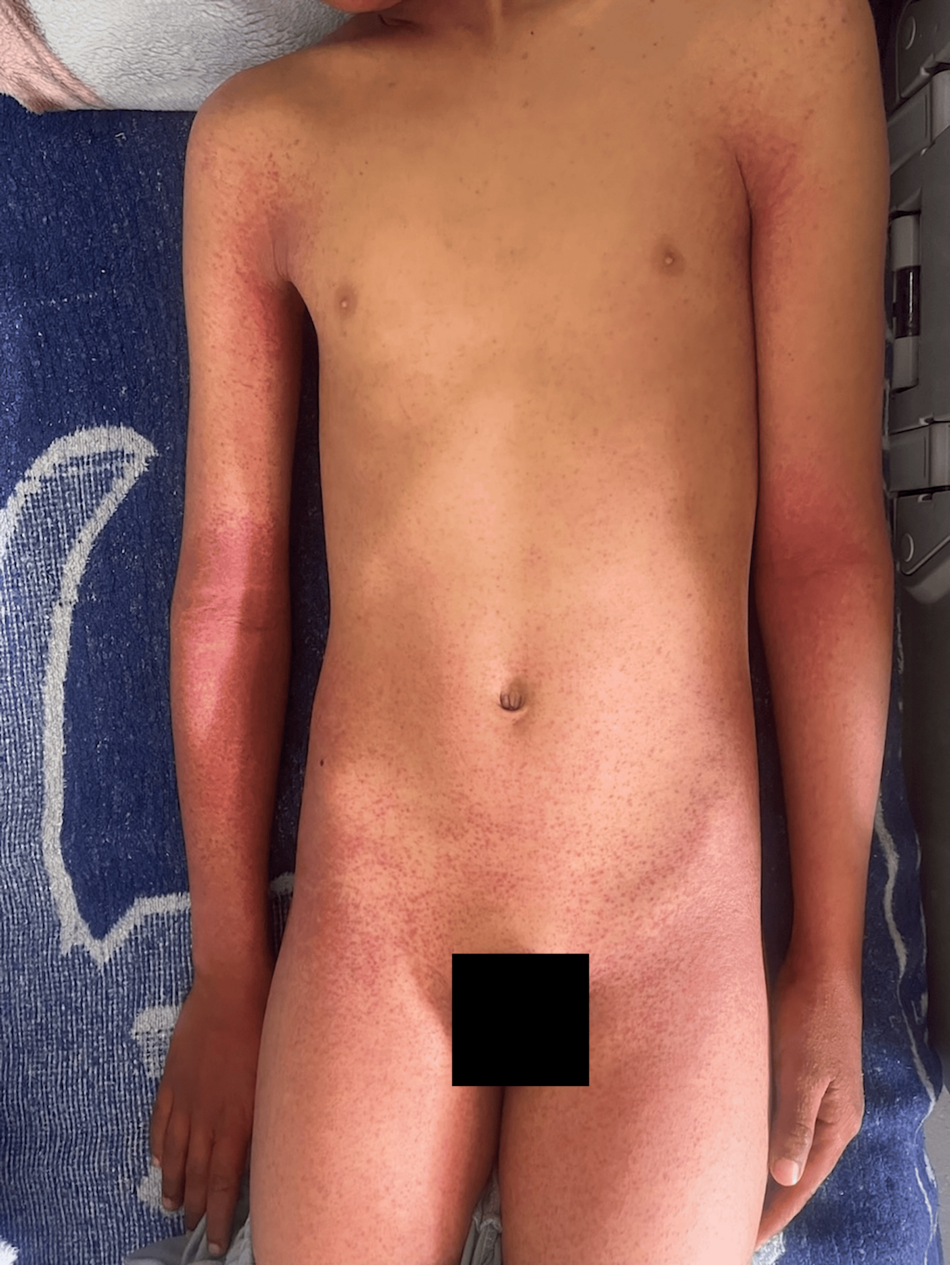
Purpuric lesions more pronounced over the elbow folds.

**Figure 2:**
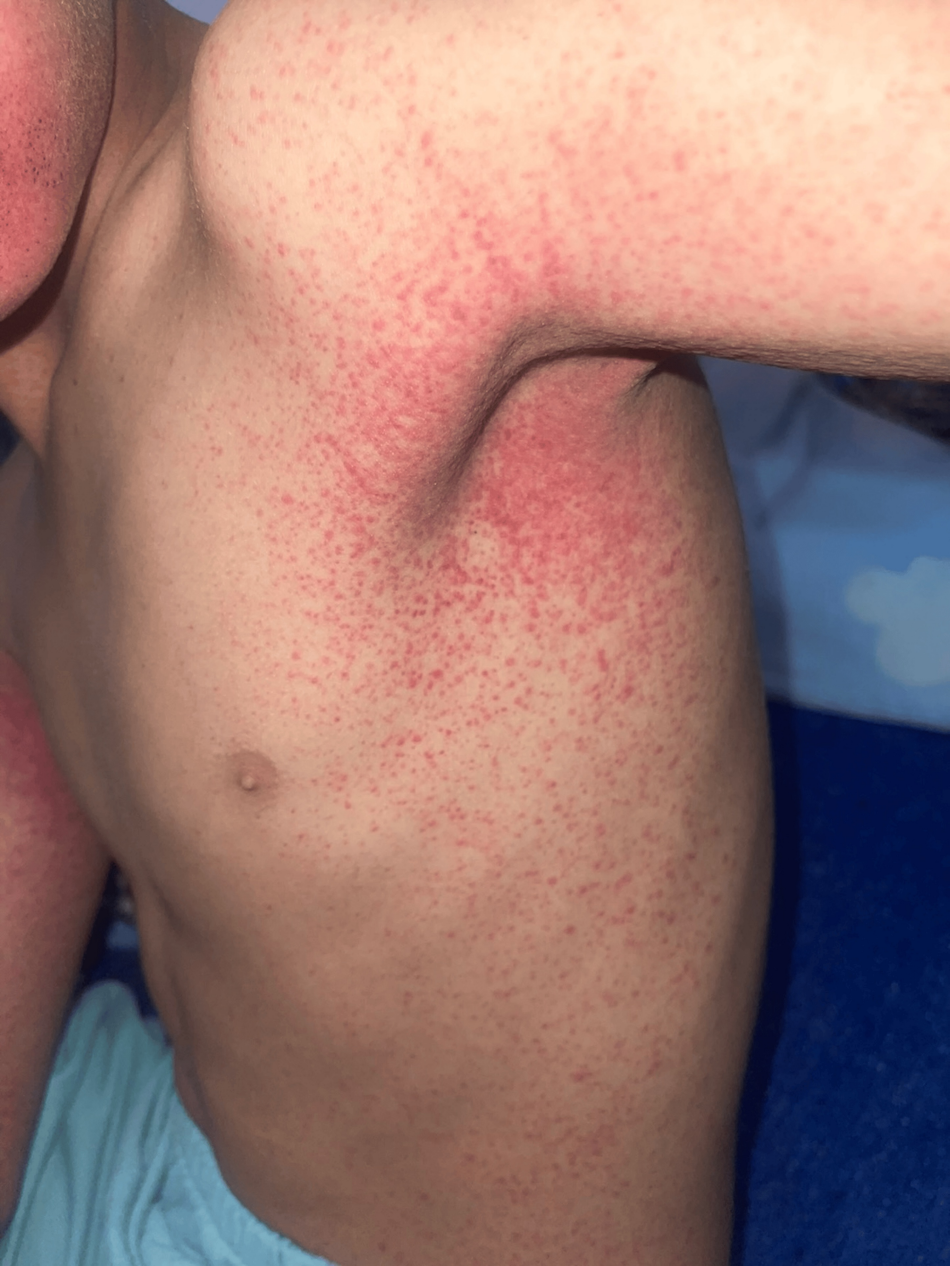
Purpuric lesions more pronounced in the axillary folds.

**Figure 3:**
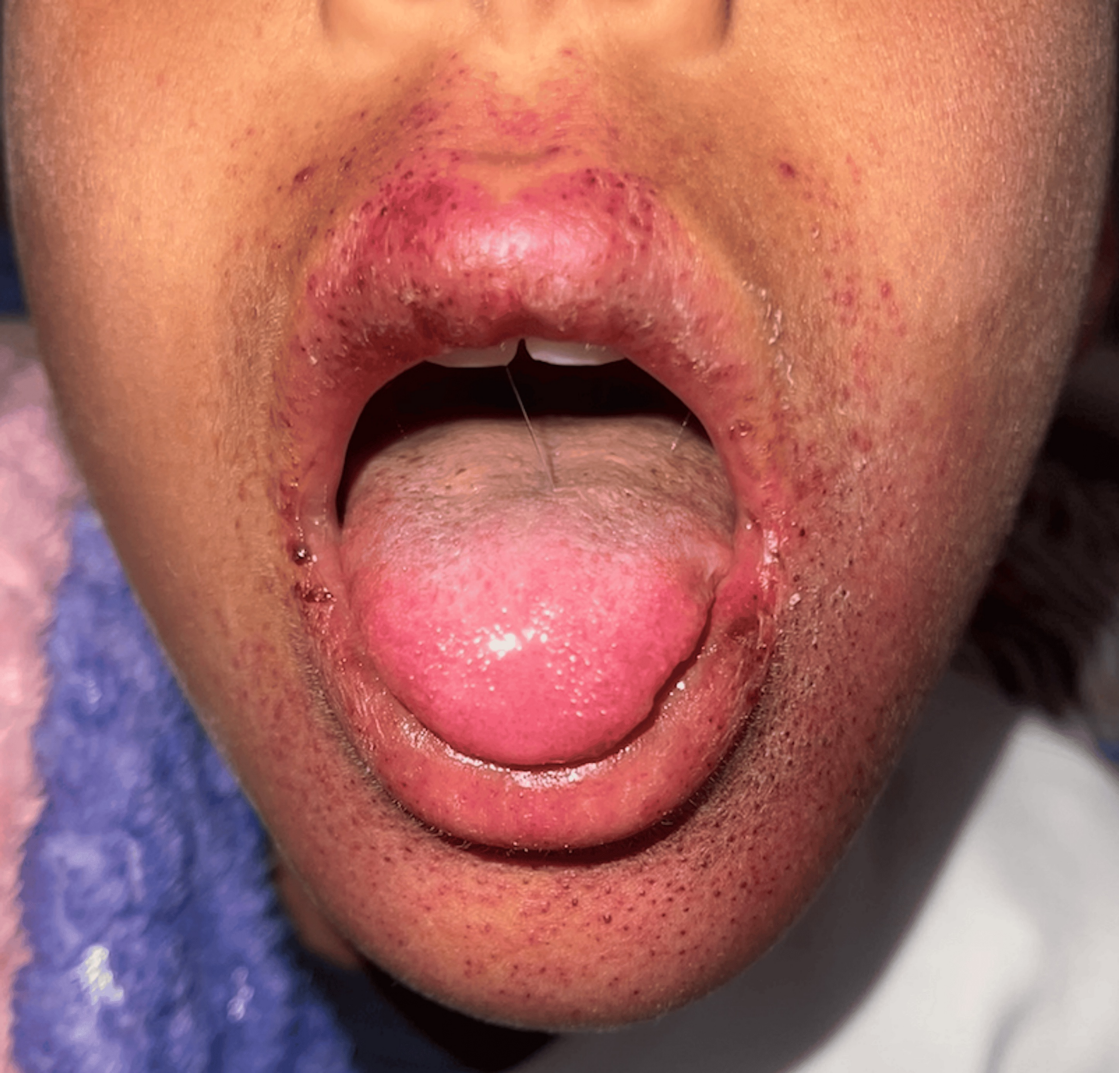
Maculo-petechial enanthem.

An 11-year-old child with no significant past medical history presented to the pediatric emergency department with a purpuric rash that had been evolving for six days, accompanied by mild febrile sensations and otherwise in good overall condition. Physical examination revealed a child who was fully conscious with a mild temperature of 38 °C. Dermatologic examination revealed widespread petechial purpuric lesions, more pronounced in the periflexural regions, including the axillary and elbow folds (Figures 1, 2). Oral mucosal examination showed a maculo-petechial enanthem (Figure 3).

Laboratory investigations demonstrated leukopenia with a white blood cell count of 2,930/mm³, lower than the normal reference range of 4,000-10,000/mm³, and neutropenia with a neutrophil count of 990/mm³, lower than the normal range of 1,500-7,500/mm³. The platelet count was 162,000/mm³, within the normal reference range (150,000-400,000/mm³), indicating the absence of thrombocytopenia. C-reactive protein was at 12 mg/L, mildly elevated from the normal reference value of below 5 mg/L. All other laboratory parameters were within normal limits.

Given the atypical distribution of the purpuric lesions, serologic testing for parvovirus B19 was performed. The test results returned positive for IgM antibodies with a level of 79.6 IU/mL, exceeding the positivity threshold of approximately 25 IU/mL. In contrast, IgG antibodies were negative at 1.8 IU/mL, with values below 2 IU/mL considered negative. This serologic profile was consistent with a recent primary parvovirus B19 infection.

The patient received symptomatic treatment, and clinical as well as laboratory parameters spontaneously improved within one week.

## Discussion

Parvovirus B19 is a major human pathogen responsible for a wide spectrum of clinical manifestations, predominantly affecting pediatric populations [3]. Although it is classically recognized as the etiological agent of erythema infectiosum (fifth disease), a generally benign childhood illness, the clinical spectrum of parvovirus B19 infection extends well beyond this conventional framework [6].

Among its less typical presentations, parvovirus B19 may cause papular-purpuric eruptions involving the hands and feet, known as the papular-purpuric “gloves and socks” syndrome. This manifestation is characterized by an abrupt onset with rapidly progressive, symmetrical edema and erythema of the distal extremities, frequently associated with petechial or purpuric lesions. The eruption typically shows a sharp demarcation at the wrists and ankles and is often accompanied by intense pruritus [7].

The differential diagnosis of pediatric purpura is broad and includes IgA vasculitis, thrombocytopenic purpura, septicemia-related purpura, and viral exanthems. IgA vasculitis typically presents with palpable purpura of the lower limbs, often with systemic involvement, whereas parvovirus B19-related purpura is more often periflexural or acral and associated with mild or absent systemic symptoms. Normal platelet counts are essential to exclude thrombocytopenic etiologies.

In our case, the clinical presentation differed from the classical dermatological manifestations usually associated with parvovirus B19 infection. The patient exhibited a widespread petechial purpura with a striking predominance in periflexural areas, particularly the axillary and elbow folds, which represents an unusual distribution pattern. Unlike the typical acral involvement observed in papular-purpuric “gloves and socks” syndrome, the lesions in our patient were not limited to distal extremities and lacked the sharply demarcated acral pattern. According to literature, the underlying pathophysiology of this unusual distribution could be the result of an exaggerated immune-mediated response to viral antigens rather than direct cytopathic effects [7].

Atypical exanthems associated with parvovirus B19 infection remain insufficiently characterized in the literature, with most data derived from isolated case reports and small series. In pediatric populations, these atypical presentations often begin with the classic “slapped cheek” appearance, followed by two main morphological patterns: a maculopapular eruption with diffuse petechiae predominantly affecting the trunk and lower limbs, or a petechial eruption confined to the extremities. Unusual distributions have also been described, including periflexural purpura mimicking Baboon syndrome [8].

The diagnosis of atypical presentations relies primarily on serological testing. Evidence of recent parvovirus B19 infection is usually based on the detection of specific IgM antibodies and a significant increase (fourfold) in IgG titers between acute and convalescent samples. In certain situations, polymerase chain reaction (PCR) testing may be useful to confirm the diagnosis, particularly when clinical findings are atypical [8,9]. The laboratory findings in our patient supported the diagnosis of acute parvovirus B19 infection. Serology for parvovirus B19 was performed and returned positive, showing IgM antibodies at a level of 79.6 IU/mL, exceeding the positivity threshold of approximately 25 IU/mL, indicating a recent primary infection.

Early recognition of atypical cutaneous manifestations is essential to ensure appropriate management and to prevent unnecessary investigations or inappropriate treatments. In most cases, parvovirus B19 infection remains self-limiting and does not require specific antiviral therapy. Management is primarily symptomatic, including antipyretics for fever and antihistamines for pruritus when present [10]. Nonsteroidal anti-inflammatory drugs may be prescribed for patients with significant arthralgia. Severe hematological complications, such as transient aplastic crisis, may necessitate red blood cell transfusions, while chronic red cell aplasia may require intravenous immunoglobulin therapy. Intravenous immunoglobulins have also been reported as beneficial in severe or persistent cases. To this day, despite ongoing research efforts, no vaccine against parvovirus B19 is currently available for clinical use [2,10]. In our case, the patient received symptomatic treatment, and clinical as well as laboratory parameters spontaneously improved within one week.

Overall, our observation further contributes to the growing body of literature highlighting the polymorphic nature of parvovirus B19 cutaneous manifestations. Recognition of such atypical presentations is particularly important in pediatric practice, as it may prevent unnecessary investigations, invasive procedures, or inappropriate treatments. Clinicians should consider parvovirus B19 infection in the differential diagnosis of periflexural purpura and unexplained petechial eruptions, especially when accompanied by mild systemic symptoms and transient cytopenias.

## Conclusions

In conclusion, clinicians should consider the following: (1) parvovirus B19 infection may present with diverse cutaneous manifestations, including atypical forms beyond the classic erythema infectiosum; (2) purpuric, acral, or periflexural eruptions may mimic inflammatory or vascular dermatoses, which can complicate the clinical diagnosis; (3) parvovirus B19 should be considered in the differential diagnosis of periflexural purpura, particularly in pediatric patients; and (4) early recognition is important to avoid unnecessary investigations, as management is usually symptomatic in most cases.
